# Adaptable Automation Transparency: Should Humans Be Provided Flexibility to Self-Select Transparency Information?

**DOI:** 10.1177/00187208251349269

**Published:** 2025-06-15

**Authors:** Monica Tatasciore, Laura Bennett, Vanessa K. Bowden, Jason Bell, Troy A. W. Visser, Ken McAnally, Jason S. McCarley, Matthew B. Thompson, Christopher Shanahan, Robert Morris, Shayne Loft

**Affiliations:** 1 2720 The University of Western Australia, Australia; 2 1974 University of Queensland, Australia; 3 2694 Oregon State University, USA; 4 Murdoch University, Australia; 5 685855 Centre for Biosecurity and One Health, Harry Butler Institute, Murdoch University, Australia; 6 2222 Defence Science and Technology Group, Australia

**Keywords:** adaptable automation transparency, uninhabited vehicle control, decision support aids, decision risk, human–automation teaming

## Abstract

**Objective:**

We examined whether allowing operators to self-select automation transparency level (adaptable transparency) could improve accuracy of automation use compared to nonadaptable (fixed) low and high transparency. We examined factors underlying higher transparency selection (decision risk, perceived difficulty).

**Background:**

Increased fixed transparency typically improves automation use accuracy but can increase bias toward agreeing with automated advice. Adaptable transparency may further improve automation use if it increases the perceived expected value of high transparency information.

**Methods:**

Across two studies, participants completed an uninhabited vehicle (UV) management task where they selected the optimal UV to complete missions. Automation advised the optimal UV but was not always correct. Automation transparency (fixed low, fixed high, adaptable) and decision risk were manipulated within-subjects.

**Results:**

With adaptable transparency, participants selected higher transparency on 41% of missions and were more likely to select it for missions perceived as more difficult. Decision risk did not impact transparency selection. Increased fixed transparency (low to high) did not benefit automation use accuracy, but reduced decision times. Adaptable transparency did not improve automation use compared to fixed transparency.

**Conclusion:**

We found no evidence that adaptable transparency improved automation use. Despite a lack of fixed transparency effects in the current study, an aggregated analysis of our work to date using the UV management paradigm indicated that higher fixed transparency improves automation use accuracy, reduces decision time and perceived workload, and increases trust in automation.

**Application:**

The current study contributes to the emerging evidence-base regarding optimal automation transparency design in the modern workplace.

## Introduction

Contemporary work environments are progressively integrating decision-aid automation that provides human operators with information, recommendations, or predictions (O’Neill et al., 2022). Although these aids have improved decision making in work domains such as defence, finance, healthcare, and aviation (NASEM & National Academies of SciencesEngineering, 2022) they may not be perfectly reliable, potentially resulting in automation *misuse* (accepting incorrect advice), or *disuse* (rejecting correct advice; Lee & See, 2004; Parasuraman & Riley, 1997). To appropriately calibrate their trust in advice, human operators need to be able to predict how an automated aid will likely perform under varying task conditions (Carter et al., 2024; Lee & See, 2004). Automation *transparency* (historically referred to as *visibility*, Dorneich et al., 2017; or *observability*, Christoffersen & Woods, 2002) can enhance trust calibration by helping the human understand the reasoning underlying automated advice and predicted outcomes if that advice is followed (Chen et al., 2014). The National Academies of Sciences, Engineering, and Medicine defined transparency as providing ‘a real-time understanding of the actions of the AI system’ (NASEM & National Academies of SciencesEngineering, 2022, p. 31). Transparency design is often guided by the Situation Awareness Agent-Based Transparency (SAT) model (Chen et al., 2014; also see Lyons, 2013) that proposes three levels of automation transparency: Level 1 – purpose and intention, Level 2 – rationale behind decision advice, and Level 3 – projected task outcomes if advice is followed. Whilst the SAT model is often used, transparency design is influenced by the task domain in which automation is applied (Skraaning & Jamieson, 2021; Van de Merwe et al., 2022, 2024).

Increased transparency has been shown to improve the accuracy of automation use (i.e. correctly accepting/rejecting advice) across multiple task domains, typically without impairing decision time or workload (see Bhaskara et al., 2020; Sargent et al., 2023; Van de Merwe et al., 2022 for reviews). In uninhabited vehicle (UV) management tasks (of interest in the current study and to Defence), increased transparency leads to more accurate automation use (e.g. Bhaskara et al., 2021; Gegoff et al., 2025; Mercado et al., 2016; Stowers et al., 2020; Tatasciore et al., 2023; Tatasciore & Loft, 2024) and greater perceived trust and usability (e.g. Tatasciore & Loft, 2024; Tatasciore & Loft, 2025), without costs (or sometimes even a benefit) to decision time or workload (Bhaskara et al., 2021; Mercado et al., 2016; Tatasciore et al., 2024; Tatasciore & Loft, 2025). Increased transparency can also mitigate the disuse associated with low-reliability automation (Gegoff et al., 2024).

However, increased transparency can lead to an over-tendency toward agreeing with automated advice, resulting in no improvement or reduced correct rejection rates (i.e. correctly rejecting incorrect advice; Bhaskara et al., 2021; Tatasciore et al., 2023; Tatasciore & Loft, 2024; Tatasciore et al., 2024). Mosier and Skitka (1996) proposed that humans can misuse automated advice due to ‘automation bias’ – defined as humans using the outcome of the decision aid ‘as a heuristic replacement for vigilant information seeking and processing’ (p. 205). Further, transparency does not appear to mitigate the costs of adverse contextual factors on the accuracy of automation use, such as nonautomated concurrent task demands (Tatasciore et al., 2023) or high time pressure (Tatasciore & Loft, 2024). These findings suggest that participants in these prior studies did not optimally use high transparency information to verify automated advice, in line with more general findings of human inefficiency in integrating task information with automated advice (e.g. Bartlett & McCarley, 2017, 2021; Boskemper et al., 2022; Tikhomirov et al., 2023), such as operators deferring to advice uncritically (for human computational modelling; see Strickland et al., 2021, 2023).

To minimise decision errors arising from inappropriate reliance on transparent automation, it may be beneficial to allow operators to self-select the transparency they require (low vs. high) on a case-by-case basis. We refer to this novel concept as *adaptable transparency*. As detailed below, purposively selecting high transparency information may increase the probability of operators thoroughly processing that high transparency information. The current study examined whether adaptable transparency could improve the accuracy of automation use, as compared to nonadaptable (hereafter referred to as ‘fixed’) low and high transparency, in a UV management task. Furthermore, we examined whether perceived task difficulty and decision risk (i.e. incorrect decision consequence) impacted whether participants selected high transparency.

### Adaptable Transparency

Adaptable transparency provides humans with flexibility to self-select the level of transparency they require. Studies indicate that humans prefer to minimise cognitive effort to make decisions (e.g. cognitive-miser hypothesis; Wickens & Hollands, 2000), often accepting and actioning automated advice despite available disconfirming evidence (Layton et al., 1994; Molloy & Parasuraman, 1996; Skitka et al., 1999). Purposefully selecting high transparency could increase the probability that operators engage with and thoroughly process transparency information to make more informed decisions (Miller, 2023; Pescetelli et al., 2021; Vered et al., 2020). This conjecture follows from theories of human supervisory monitoring (Moray, 2003; Senders, 1983), and attentional allocation (Steelman et al., 2011; Wickens et al., 2015) which propose that the probability of attending to information, and the depth of subsequent information processing, is driven by the *perceived expected value* of that information in relation to task goals (i.e. individuals seek out information they believe has higher value). It follows then that if operators choose high transparency, they perceive that high transparency information is of significant value and thus may be more likely to thoroughly process raw task inputs to verify automated advice (Pescetelli et al., 2021). On this basis, adaptable transparency may improve automated advice use (e.g. including improved correct rejection rates) and increase perceived system usability and trust, compared to fixed low and high transparency.

We are only aware of one study that has examined some form of what we refer to in this paper as adaptable transparency. A study by Vered et al. (2020) compared the effects of demand-driven transparency (participants requested transparency information as needed) and sequential transparency (participants stepped through all transparency information in a fixed order) in a UV task. Participants in the demand-driven transparency condition selected high transparency on 68% of trials, made faster decisions, and reported higher trust than those who received sequential transparency, whilst maintaining a similar level of decision-accuracy.

While adaptable transparency may lead to benefits over fixed transparency designs, there are several potential constraints. In operational contexts, high transparency selection may produce display clutter (Moacdieh & Sarter, 2015) or take significant time to populate on displays. In our pilot study, when provided adaptable transparency, participants indicated a higher reluctance to select high transparency than we had expected. Thus, we applied neither constraint (clutter, time delay) here so as not to discourage high transparency selection. The observed reluctance to select high transparency may reflect that higher transparency selection requires more information to be subsequently processed. When using adaptable automation, participants may decide to withhold higher transparency when deemed unnecessary to minimise cognitive effort/load (Wickens & Hollands, 2000), and be more likely to select high transparency when they have a lower perceived ability to make a correct decision without high transparency (Hutchinson et al., 2023). We thus anticipated that participants would select high transparency based on perceived task difficulty. However, limitations in meta-cognition may mean that participants may not always be able to accurately estimate task difficulty or monitor their own capability, resulting in sub-optimal case-by-case transparency selection (Flavell, 1979; Osman, 2010). The benefits of adaptable transparency are likely to be partly dependent on participants being able to accurately monitor their task environment and their own task performance to decide when they most require the higher transparency.

The current study examined whether adaptable transparency could improve the accuracy of automation use compared to fixed low and high transparency in a UV management task. We measured high transparency selection as a function of perceived task difficulty. Purposefully selecting high transparency should increase the probability that operators thoroughly process that high transparency information, resulting in more accurate automation use compared to than seen with fixed transparency. We also expected that perceived trust and automation usability would be higher with adaptable compared to fixed transparency. However, we expected workload would increase with adaptable transparency compared to fixed transparency due to the cognitive capacity required to decide when to select high transparency (see Chen et al., 2017; Greenlee et al., 2022; Thropp et al., 2018).

### Decision Risk

Operators may also select transparency based on contextual factors such as decision risk. Decision risk refers to the consequences of an incorrect decision (Knighton, 2004), and in Defence contexts potentially ranges from damaged equipment (lower risk) to human fatalities (higher risk). There is evidence that automation verification (Loft et al., 2023; Satterfield et al., 2017) and the subsequent accuracy of automation use (Loft et al., 2023; Perkins et al., 2010; Satterfield et al., 2017) can increase with higher decision risk.

We examined the impact of decision risk on the accuracy of automation use and decision time but were primarily interested in the impact of decision risk on transparency selection. Participants were informed that choosing the incorrect UV for a mission would lead to a lower performance-based monetary bonus, particularly for higher-risk missions. This method was used by Loft et al. (2023), who reported that high decision risk increased automated advice verification and improved correct rejection rates in a similar UV management task. We predicted that high transparency would be selected more frequently under high decision risk. To the extent higher transparency is selected as a function of decision risk, the accuracy of automation use may improve when risk is higher.

### Current Study

An adapted version of our UV management task (Tatasciore et al., 2023, 2024; Tatasciore & Loft, 2024, 2025) was used. On each discrete trial, participants selected one UV (out of two options) to complete the mission, after receiving advice from a *Recommender* system (decision aid). The optimal UV was determined by relative UV capabilities (e.g. fuel consumption), capability importance weightings, and environmental factors impacting those capabilities.

A 3 × 2 within-subjects design was used with three levels of transparency (fixed low, fixed high, adaptable) and two levels of decision risk (low, high). Under the adaptable transparency condition, participants selected which level of transparency they preferred, low or high, on a trial-by-trial basis. The SAT model was used to guide the transparency designs, in consultation with the Australian Defence Science and Technology Group. Low transparency broadly equated to SAT Level 1 (e.g. information about the automation’s goals/intent) and displayed how the Recommender assessed the importance of UV capability weightings. High transparency condition broadly equated to SAT Level 1 + 2+3 by additionally presenting which UV the Recommender considered to be better on each capability, and by displaying information on *how* the Recommender calculated UV capabilities, including which environmental factors it considered and their projected impact on each UV capability.

## Experiment 1

### Participants

Participants were 73 (47 females, 26 males; *M* = 25.12 years) undergraduate students who received course credits and a performance-based bonus of up to AUD30. An a priori power analysis revealed that a minimum sample size of 73 would provide power of 0.95 to detect a small to medium effect of increased fixed transparency (low vs. high) on the accuracy of automation use. This power calculation was based on effect size data (*d* = .43) from the outcomes of our prior studies that manipulated fixed transparency within-subjects (e.g. Tatasciore & Loft, 2024). This research complied with the American Psychological Association Code of Ethics and was approved by the Human Resource Ethics Office at The University of Western Australia. Informed consent was obtained from each participant.

### Uninhabited Vehicle Management Task

**Figure 1.**
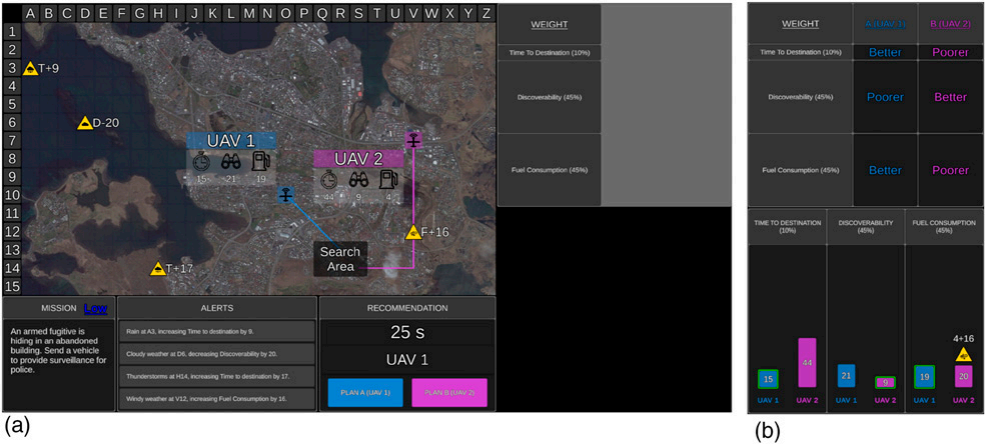
The uninhabited vehicle management task with low (a) and high (b) transparency. *Note.* The mission, tactical map, alerts, and recommendation displays are identical regardless of the transparency condition. Here, the Recommender has correctly advised UAV 1 as Plan A. Transparency information is presented in the table (top right) and graph (high transparency only) displays. The table display presents how the Recommender evaluated the importance of UV capabilities (larger rows = more important). High transparency (b) also presents which UV the Recommender considered to be better and poorer on each capability, and a graph display that illustrated how the Recommender calculated capability scores including which factors it took into consideration and the impact value of relevant factors. The bar of the UV that the Recommender considered to be better was outlined green.

The UV management task was presented on a single desktop monitor (Figure 1) and comprised 150 trials (missions). Participants were asked to choose the optimal UV to complete each mission. The tactical map displayed an aerial image of either rural, coastal, or urban areas. On the tactical map were the search area and two UVs (aerial [UAV]; ground [UGV]; surface [USV]), which were arbitrarily labelled 1 or 2. Projected lines from each UV depicted their route to the search area. Mission statements were included in the mission window for face validity but were irrelevant to UV selection. Decision risk was presented in the mission window, with ‘Low’ in blue for low-risk missions and ‘High’ in red for high-risk missions. Participants were informed that choosing the incorrect UV would lead to a lower performance-based monetary bonus, particularly if they choose the incorrect UV on high-risk missions (Loft et al., 2023).

The optimal UV for each mission was determined based on UV capabilities, capability importance weightings, and environmental factors impacting UV capabilities. UV capabilities included time to destination (timer symbol on tactical map) representing the time required to reach the search area (lower score = quicker); discoverability (binocular symbol) representing how discoverable UVs were by third parties (lower = less discoverable); and fuel consumption (fuel gauge symbol) representing fuel required to reach the search area (lower = less fuel required).

Capability weightings for each UV were displayed in the weightings window as percentages. One of five weighting sets was chosen for each mission, with sets differing in difficulty. Harder weighting sets involved two equally weighted capabilities (e.g. 40%, 40%, and 20%), challenging participants to determine which UV scored lower on two capabilities. Easier weighting sets included one highly weighted capability (e.g. 80%, 10%, and 10%) and thus it was only necessary to consider which UV scored lower on that capability.

Environmental factors were presented as yellow factor symbols on the tactical map. Four factors were presented during each mission, with one to three factors being relevant to UV capabilities. Relevant factor symbols appeared on the path of a UV, whereas irrelevant factors did not and could be ignored. Factor symbols conveyed the type of factor (e.g. rain), the capability impacted (T = time to destination, D = discoverability, F = fuel consumption), the direction (+= positive, − = negative), and the level of its impact. The alerts window also provided messages regarding factors and their impact.

The Recommender advised the optimal UV as Plan A and the other as Plan B (Recommendation window; Figure 1), after considering UV capabilities, their weightings, and environmental factors impacting capabilities. Participants were required to either agree with the Recommender and select Plan A or to instead choose Plan B. Feedback was provided after each mission regarding the accuracy of the Recommender and the participant’s decision.

Participants were aware of the Recommender’s goals and intent, and further, low transparency information was presented in the weightings window, demonstrating how the Recommender evaluated the importance of UV capabilities, with larger table rows for capabilities with higher weightings (Figure 1(a)).

With high transparency, the table display also presented which UV the Recommender considered to be ‘better’ and ‘poorer’ on each capability (Figure 1(b)). In addition, high transparency included a display (Figure 1(b)) of three bar graphs, one for each capability. The graph illustrated how the Recommender calculated the score for each capability, including which factors it considered. When the Recommender considered a relevant factor, the factor symbol appeared on top of the relevant bar on the graph. Additionally, above the factor symbol was the impact value which the Recommender added or subtracted from the original capability score. The resulting calculated score was presented within the respective bar. As lower scores were considered better, shorter bars represented better capability. High transparency therefore displayed information regarding the projected consequences of variability in the task environment, and thus projected outcomes if its advice was actioned. A green border was also placed around the bar of the UV that the Recommender considered to be better on each capability. The bars for Plan A were always presented on the left-hand side and coloured blue.

The Recommender was 80% reliable, advising the incorrect UV as Plan A on 20% of trials. Participants were informed that ‘*The Recommender is a highly reliable system, but it is not perfect and as such its recommendation may not be the most optimal plan 100% of the time*’. When the Recommender provided incorrect advice, it either missed a relevant factor, miscalculated the impact of a relevant factor, or a combination of the two errors across different relevant factors.

With high transparency, if the Recommender missed a relevant factor, the factor symbol was missing from the graph display, and the original score was incorrectly presented for the UV capability affected. If the Recommender miscalculated the impact of a relevant factor, the factor symbol was presented on the graph, but the score that it added or subtracted was incorrect, resulting in an incorrect final score. Further, the green border may have been placed around the incorrect bar on the graph. Given these errors in the table display, the UV that the Recommender considered to be ‘better’ and ‘poorer’ on each capability may also have been incorrect.

On 10% of the ‘reliable’ trials, the Recommender advised the correct UV as Plan A but still made one of the above errors. However, these errors were not great enough to cause an incorrect UV recommendation. These trials were included to discourage participants from immediately selecting Plan B as soon as they noticed any error made by the Recommender (e.g. Gegoff et al., 2024, 2025; Tatasciore et al., 2023; Tatasciore & Loft, 2024).

A total of 150 mission trials were completed, split into two blocks: fixed (100 trials, with a minimum break of 60 s after 50 trials) and adaptable (50 trials). During the fixed block, 50 low and 50 high transparency trials were randomly presented (i.e. low and high transparency trials intermixed). During the adaptable block, participants could choose the transparency they required for each mission. Three sets of 50 mission trials were designed to ensure similar task difficulty (i.e. capability weightings, relevant environmental factors, and type of errors) across each set. The presentation order of mission sets and their assignment to blocks (fixed or adaptable), block order (i.e. adaptable first or second), and the assignment of mission risk (low, high) to specific missions was counterbalanced across participants.

#### Missions

**Figure 2.**
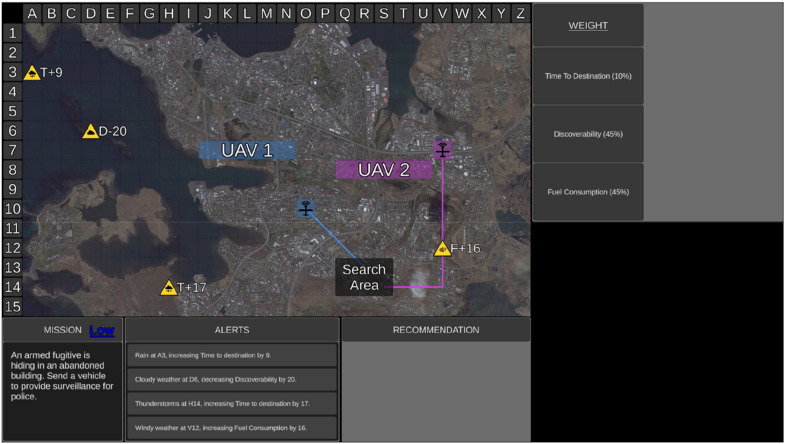
The uninhabited vehicle management task during the Surveillance Phase. *Note*. During the surveillance phase UV capability scores were blanked and the Recommender’s advice and transparency information were not presented.

**Figure 3.**
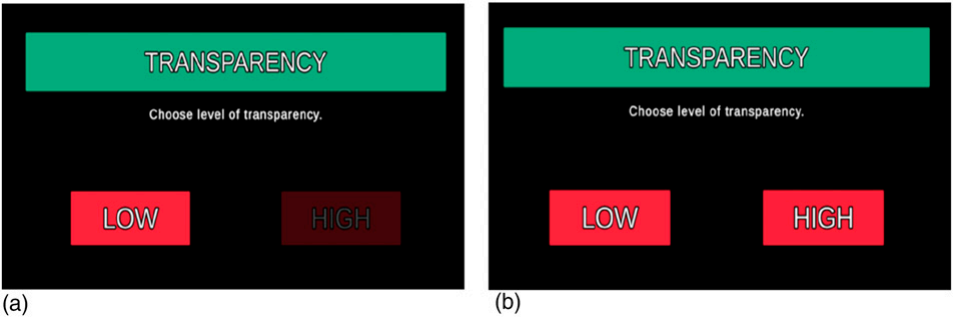
The transparency selection display for fixed (a) and adaptable (b) transparency.

Each mission began with a 6 s duration *surveillance phase* (Figure 2) to allow participants to review mission risk, capability weightings, and relevant environmental factors to assess the mission difficulty. During this phase, the Recommender’s advice and transparency information were not presented, and UV capability scores were blanked. Following the surveillance phase, participants rated mission difficulty. In the fixed block, participants then confirmed the preassigned transparency which varied on a trial-by-trial basis (Figure 3(a)). In the adaptable block, participants chose the transparency (low or high) they required to complete the mission (Figure 3(b)).

Following this, the *decision phase* began, lasting 24 s (maximum duration). At the onset of the decision phase, UV capability scores, the Recommender’s advice, and transparency information were presented.

### Measures

#### Accuracy of Automation Use

Hit rate was the proportion of trials in which participants correctly chose Plan A. Correct rejection rate was the proportion of trials in which participants correctly selected Plan B when Plan A was incorrect. The Signal Detection parameter *d′* assessed sensitivity to discriminate Plan A from B. Parameter *c* assessed participants’ bias towards agreeing with the automation (negative values = greater bias). To account for extreme hit or false alarm values (0 or 1), rates of 0 were adjusted to 0.5/*n,* and rates of 1 were adjusted to (*n*-*0.5)/n*, where *n* represented the number of signal (Plan A correct) or noise (Plan B correct) trials (Macmillan & Kaplan, 1985). Decision times were based on correct decision trials only.

#### Perceived Difficulty

Perceived difficulty was measured after each mission surveillance phase, hence before each mission decision phase. Participants responded to the item *‘Rate this mission’s difficulty’* on a 10-point scale, ranging from 1 (easy) to 10 (very difficult).

#### Workload

Workload was measured using the National Aeronautics and Space Administration Task Load Index (NASA-TLX; Hart & Staveland, 1988). Participants rated workload from 0 to 100 on six subscales (temporal demands, physical demands, mental demands, frustration, performance, effort), and completed pairwise comparisons to indicate which subscales contributed most to workload (i.e. weighted subscale scores). Weighted subscale scores were multiplied by the rating for each subscale and divided by 15 to calculate an overall task load index score. Workload could range from 0 (low workload) to 100 (high workload).

#### Trust

Trust was measured using a modified version of the six-item questionnaire by Merritt (2011). Participants rated items such as *‘I believe the Recommender is a competent performer’*, using a 5-point scale, ranging from 1 (strongly disagree) to 5 (strongly agree). Items are presented in Supplementary Materials.

#### System Usability Scale

Perceived usability of automation was assessed using a modified version of the 10-item System Usability Scale (SUS; Brooke, 1996). Responses to items such as *‘I think that I would like to use the Recommender frequently’* were indicated on a 5-point scale, ranging from 0 (strongly disagree) to 4 (strongly agree). Some items were reverse-scored and then all items added and multiplied by 2.5 to yield a score from 0 to 100. Items are presented in Supplementary Materials.

### Procedure

Participants viewed a 25-min audio-visual training presentation detailing how to complete the task manually, followed by 20 manual practice trials. Next, participants viewed a 20-min audio-visual presentation explaining the first block of experimental trials (adaptable or fixed). Following the first block, participants completed the NASA-TLX (subscale ratings), Trust, and SUS questionnaires. They then viewed a 5-min audio-visual presentation explaining the second block. Following the second block, participants completed the NASA-TLX (subscale ratings and subscale pairwise comparison ratings), Trust, and SUS questionnaires. The total experiment duration was 2.5 h. Participants were encouraged to take breaks throughout the experiment (i.e. after training presentations, blocks of trials, and questionnaire administration).

## Results

Manual training descriptive statistics, as a function of decision risk, are presented in Table 1. They show no differences on outcome variables (smallest *p* = .12).

**Table 1.** Means and Standard Deviations (Presented in Parentheses) for Manual Training Trials as a Function of Decision Risk.

	Low Decision Risk	High Decision Risk
UV selection accuracy	.80 (.20)	.84 (.16)
Correct decision time	17.26 s (3.79 s)	17.48 s (3.88 s)

*Note.* s = seconds.

**Figure 4.**
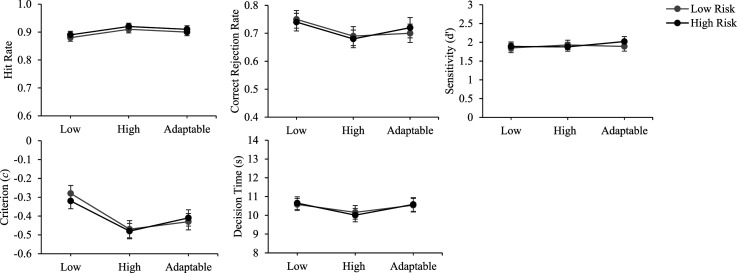
Accuracy of automation use and decision time as a function of transparency and decision risk. *Note.* Error bars represent the standard error of the mean. Low = low transparency; high = high transparency; adaptable = adaptable transparency.

Descriptive statistics for test trials as a function of transparency level and decision risk are presented in Table 2 and illustrated in Figure 4. We ran 3 Transparency (fixed low, fixed high, adaptable) × 2 Decision risk (low, high) × 2 Transparency Presentation Order (fixed first, adaptable first) mixed ANOVAs for accuracy of automation use and decision time. Main effects of transparency were followed up with planned paired samples *t*-tests that directly paralleled our research questions by comparing fixed low and high transparency, fixed low and adaptable transparency, and fixed high and adaptable transparency.

**Table 2.** Means and Standard Deviations (Presented in Parentheses) for Test Trials as a Function of Transparency and Decision Risk.

	Fixed Low Transparency		Fixed High Transparency		Adaptable Transparency	
	Low Decision Risk	High Decision Risk	Low Decision Risk	High Decision Risk	Low Decision Risk	High Decision Risk
Hit rate	.88 (.11)	.89 (.11)	.91 (.11)	.92 (.10)	.90 (.11)	.91 (.11)
CR	.75 (.27)	.74 (.27)	.69 (.29)	.68 (.27)	.70 (.28)	.72 (.31)
Sensitivity (*d′*)	1.85 (1.07)	1.89 (1.03)	1.93 (1.07)	1.88 (1.01)	1.89 (1.07)	2.02 (1.14)
Criterion (*c*)	−.28 (.36)	−.32 (.35)	−.47 (.39)	−.48 (.35)	−.43 (.37)	−.41 (.37)
DT (s)	10.58 (2.72)	10.65 (2.89)	10.16 (3.10)	10.01 (3.03)	10.54 (3.04)	10.58 (3.12)
High transparency selection	-	-	-	-	.37 (.36)	.40 (.37)

*Note.* CR = correct rejection; DT = correct decision time; s = seconds.

Interactions between transparency and transparency presentation order were followed up with independent samples *t*-tests that directly assessed the impact of transparency presentation order on the key aforementioned comparisons of interest. To do this, we compared fixed low and high transparency, fixed low and adaptable transparency, and fixed high and adaptable transparency, as a function of whether the conditions in each comparison had (*both*) been presented in Block 1 or Block 2 (i.e. between-participant comparisons).

Effect sizes were estimated using partial eta squared (small = 0.01, medium = 0.06, large = 0.14) for *F*-tests and Cohen’s *d* (small = 0.20, medium = 0.50, large = 0.80) for *t*-tests (Cohen, 1992).

### Adaptable Transparency Selection

During the adaptable block, participants selected high transparency on 39% of trials, with considerable variation across participants (*SD* = 0.36). Twelve participants (16.4%) never selected high transparency, four (5.5%) selected it on every trial, while the remaining 57 (78.1%) selected a mix of low and high transparency across trials. High transparency selection rates did not differ between low decision risk (*M* = .37, *SD* = .36) and high decision risk (*M* = .40, *SD* = .37) missions, *t* (72) = 1.65, *p* = .10. Missions for which high transparency (*M* = 5.54, *SD* = 1.38) was selected were a priori rated as more difficult than those for which low transparency (*M* = 3.86, *SD* = 1.39) was selected, *t* (56) = 7.76, *p* < .001, *d* = 1.03.

### Accuracy of Automation Use

*Hit Rate*. There was a main effect of transparency, *F* (2,142) = 8.41, *p* < .001, $\eta_{\rho }^{2}$ = .11. Hit rate was higher for fixed high (*M* = .92, *SD* = .10), *t* (72) = 3.68, *p* < .001, *d* = .43, and adaptable transparency (*M* = .91, *SD* = .10), *t* (72) = 2.97, *p* = .004, *d* = .35, compared to fixed low (*M* = .88, *SD* = .09). There was no difference in hit rate between fixed high and adaptable transparency, *t* < 1.

There was an interaction between transparency and order, *F* (2,142) = 6.29, *p* = .01, $\eta_{\rho }^{2}$ = .08. Table 3 indicates hit rates were higher in Block 2 compared to Block 1 for each condition, explaining the interaction between transparency and order. In Block 1, there was no difference in hit rate between fixed low and high transparency, *t* (72) = 1.15, *p* = .25. In contrast, in Block 2, hit rate was higher for fixed high compared to fixed low transparency, *t* (70) = 2.25, *p* = .03, *d* = .57. In Block 1, there was no difference in hit rate between adaptable and fixed low, *t* (71) = 1.19, *p* = .24, or fixed high transparency, *t* < 1. In Block 2, there was no difference in hit rate between adaptable and fixed low, *t* (71) = 1.29, *p* = .20, or high transparency, *t* (71) = 1.02, *p* = .31.

**Table 3.** Means and Standard Deviations (Presented in Parentheses) for Hit Rate and Correct Decision Time as a Function of Transparency and Transparency Presentation Order.

	Fixed Block 1		Adaptable Block 1	Fixed Block 2		Adaptable Block 2
	Low	High		Low	High	
Hit rate	.87 (.10)	.90 (.12)	.90 (.10)	.89 (.08)	.93 (.06)	.92 (.10)
DT (s)	11.31 (2.60)	11.20 (2.68)	11.41 (3.10)	9.91 (2.65)	8.98 (2.95)	9.71 (2.74)

*Note.* Low = low transparency; high = high transparency; DT = correct decision time; s = seconds.

There were no main effects of decision risk, *F* (1,72) = 1.75, *p* = .19, or order, *F* < 1. There was no interaction between transparency and decision risk, *F* < 1, and no three-way interaction, *F* < 1.

#### Correct Rejection Rate

There were no main effects of transparency, *F* (2,142) = 2.44, *p* = .09, decision risk, *F* < 1, or order, *F* < 1. There were no interactions between transparency and order, *F* (2,142) = 1.27, *p* = .29, transparency and decision risk, *F* < 1, and no three-way interaction, *F* < 1. As indicated in Table 2, correct rejection rates were numerically lower (poorer) for the fixed high compared to the adaptable transparency condition and were numerically highest for the fixed low condition.

#### Sensitivity

There were no main effects of transparency, *F* < 1, decision risk, *F* < 1, or order, *F* < 1. There were no interactions between transparency and order, *F* (2,142) = 2.95, *p* = .06, transparency and decision risk, *F* < 1, and no three-way interaction, *F* < 1.

#### Response Bias

There was a main effect of transparency, *F* (2,142) = 11.34, *p* < .001, $\eta_{\rho }^{2}$ = .14. Participants were more biased toward agreeing with automated advice when using fixed high (*M* = −.48, *SD* = .31), *t* (72) = 4.59, *p* < .001, *d* = .54, and adaptable (*M* = −.42, *SD* = .32), *t* (72) = 2.90, *p* = .01, *d* = .34, compared to fixed low transparency (*M* = −.30, *SD* = .30). There was no difference in response bias between fixed high and adaptable transparency, *t* (72) = 1.73, *p* = .09. There were no main effects of decision risk or order, *Fs* < 1, and no interactions between transparency and order, *F* (2,142) = 1.24, *p* = .29, or transparency and decision risk, *F* < 1. There was no three-way interaction, *F* < 1.

### Correct Decision Time

There were no main effects of transparency, *F* (2,142) = 2.31, *p* = .10, decision risk, *F* < 1, or order, *F* < 1. There was no interaction between transparency and decision risk, *F* < 1, and no three-way interaction, *F* (2,140) = 1.11, *p* = .33.

There was an interaction between transparency and order, *F* (2,142) = 29.17, *p* < .001, $\eta_{\rho }^{2}$ = .03. Table 3 indicates faster correct decisions in Block 2 compared to Block 1 across conditions, explaining the interaction between transparency and order. In Block 1 (*t* < 1) and Block 2, *t* (70) = 1.41, *p* = .16, there was no difference in decision times between fixed low and high transparency. In Block 1, there were no differences in decision times between adaptable and fixed low or high transparency, *t*s < 1. In Block 2, there were no differences in decision times between adaptable and fixed low, *t* < 1, or high transparency, *t* (70) = 1.09, *p* = .28.

### Perceived Workload, Trust, and Usability

**Figure 5.**
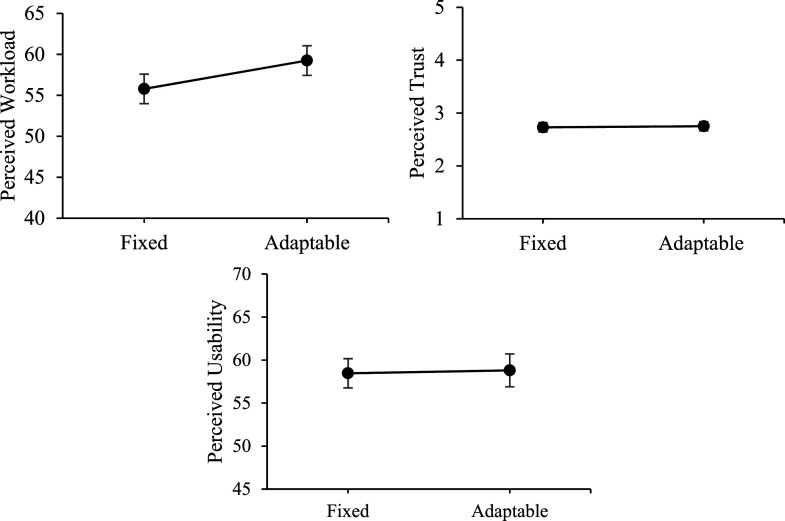
Workload, trust, and usability as a function of transparency. *Note.* Fixed = intermixed low and high transparency; Adaptable = adaptable transparency.

Descriptive statistics for perceived workload, trust, and usability as a function of transparency are presented in Table 4 and illustrated in Figure 5. For these outcome variables, we ran 2 Transparency (fixed, adaptable) × 2 Transparency Presentation Order mixed ANOVAs.

**Table 4.** Means and Standard Deviations (Presented in Parentheses) for Perceived Workload, Trust, and Usability as a Function of Transparency.

	Fixed Transparency	Adaptable Transparency
Workload	55.78 (15.37)	59.24 (15.50)
Trust	2.73 (.76)	2.75 (.74)
Usability	58.46 (14.49)	58.80 (16.30)

For perceived workload, there was a main effect of transparency, *F* (1,71) = 6.02, *p* = .02, $\eta_{\rho }^{2}$ = .08, but no main effect of order, *F* < 1, and no interaction between transparency and order, *F* (1,71) = 2.29, *p* = .13. Perceived workload was rated higher after adaptable, compared to fixed transparency blocks.

For perceived trust and usability, there were no main effects of transparency, *F* < 1, or order, *F* < 1, and no interaction, *F* < 1.

## Discussion

Experiment 1 examined whether adaptable transparency led to more accurate automation use compared to fixed low and high transparency. Further, we examined the impact of decision risk on the accuracy of automation use, and the degree to which participants selected high transparency based on perceived task difficulty and decision risk.

There was no difference in the accuracy of automation use (sensitivity) or decision time between fixed high and low transparency. This is inconsistent with our prior work using the same (or very similar) UV management tasks, which demonstrated that fixed high compared to fixed low transparency leads to more accurate automation use (e.g. Tatasciore et al., 2023; Tatasciore et al., 2024; Tatasciore & Loft, 2024; Tatasciore & Loft, 2025). Follow-up tests conducted on the transparency × transparency presentation order interaction indicated improved fixed high versus low hit rate at Block 2, but not at Block 1.

One potential explanation for the null fixed transparency findings is that, unlike previous work, the current study included a *surveillance phase*. Preexposure to mission weightings, environmental factors, and their associated impact on UV capabilities during the surveillance phase may have somehow weakened the benefit of fixed high transparency on automation use accuracy. Furthermore, the intermixed presentation of high and low transparency trials within a single block may have hindered the benefit of fixed high transparency by disrupting participant task strategies or the consistency in which they processed transparency information. It is possible that participants require consistent practice to develop strategies for effectively using the increased information provided with high transparency, such as identifying information processing shortcuts/heuristics or understanding the nuances of the presented information. Further, intermixed presentation may have introduced cognitive interference if participants were required to shift their attention set to understand the additional sources of information when increased transparency was presented (Monsell, 2003; Rogers & Monsell, 1995), and this may have hindered high transparency use strategy development. Consistent with this, in unpublished data we found that the benefit of fixed high transparency on the accuracy of automation use was greater for individuals with higher attentional control capacity (i.e. ability to maintain focus on task-relevant information whilst disengaging from distracting or less relevant information; Burgoyne & Engle, 2020). Taken together, this suggests it will be critical for future research to use eye-tracking or information unmasking techniques to examine strategy development.

In the adaptable block, high transparency was selected 39% of the time, and more often for missions perceived to be more difficult. Notably, even though there was no display clutter or time cost to selecting higher transparency, it was used on fewer than half of missions. There was no benefit to the accuracy of automation use or other outcome variables when using adaptable compared to fixed (low or high) transparency. Follow-up tests conducted on the transparency × transparency presentation order interaction indicated this was the case when assessed at both Block 1 and Block 2 (i.e. no evidence that transparency presentation order impacted the adaptable vs. fixed transparency outcomes).

Perceived workload was rated higher with adaptable compared to fixed transparency, potentially due to the cognitive demand required to select transparency information (Chen et al., 2017). This increased workload was found even though the adaptable block had fewer trials (50), and thus a shorter duration, compared to the fixed block (100).

Overall, adaptable transparency did not lead to benefits. Of course, a very real possibility is that this is because we did not observe a difference in the accuracy of automation use between fixed high and low transparency. Thus, there was no evidence that higher transparency benefited automation use, whether it was fixed or adaptable. We also found no effect of decision risk on the accuracy of automation use (or on high transparency selection rates), inconsistent with prior findings of more accurate automation use (Loft et al., 2023) with high decision risk. Considering these inconsistencies, we decided to modify the UV management task in Experiment 2 to be more in line with our previous work, before rejecting the possibility that adaptable transparency yields no benefits.

## Experiment 2

The goal of Experiment 2 was to reexamine whether adaptable transparency can improve the accuracy of automation use compared to fixed low and high transparency. We modified the UV management task design to be in line with our prior studies that have reported benefits of increased fixed transparency on automation use (Gegoff et al., 2024, 2025; Tatasciore et al., 2023, 2024; Tatasciore & Loft, 2024, 2025). Specifically, we removed the surveillance phase so that participants could no longer preassess mission weightings, environmental factors, or their impact. We separated fixed high and low transparency trials into two separate blocks of 50 trials each (i.e. not intermixed). We also removed the decision risk manipulation and perceived difficulty measure to further bring the task design in line with our prior studies.

## Method

### Participants

Participants were 88 (62 females, 24 males, 2 nonbinary; *M* = 20.02 years) undergraduate students who received course credit and a performance incentive up to AUD30. As in Experiment 1, an a priori power analysis revealed that a minimum sample size of 73 would provide power of 0.95 to detect a small to medium effect of increased fixed transparency (low vs. high) on the accuracy of automation use. Participants were unique to those that participated in Experiment 1.

### Uninhabited Vehicle Management Task

**Figure 6.**
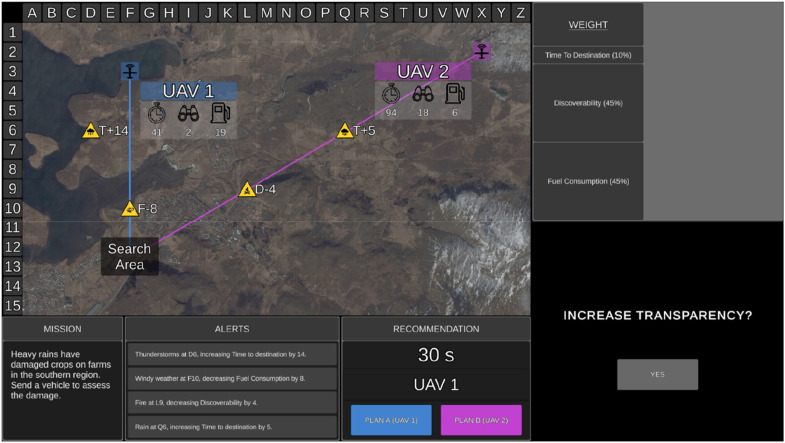
The uninhabited vehicle management task during the adaptable transparency condition in Experiment 2. *Note.* The tactical, alerts, recommendation, and weighting displays are identical to Experiment 1. Decision risk is no longer presented in the mission window. Low transparency is presented by default and high transparency can be participant-selected if required by clicking on the ‘yes’ button to increase transparency.

The UV management task was identical to that of Experiment 1 with the following exceptions. Participants completed a total of 150 trials split into three blocks of 50 trials each, with each block assigned either fixed low, fixed high, or adaptable transparency. The surveillance phase was removed and thus each trial lasted a maximum of 30 s. UV capabilities, automated advice, and transparency information were presented from the onset of each trial. During the adaptable transparency block, participants received low transparency by default and could select high transparency at any time during the trial by clicking on the ‘yes’ button (Figure 6). Decision risk was removed from the display.

The missions in the three sets of 50 trials were identical to Experiment 1. The presentation order of trial sets and their assignment to blocks (fixed low, fixed high, or adaptable) and block order (i.e. adaptable first, second, or third) was counterbalanced.

### Measures and Procedure

Accuracy of automation use and the perceived trust/usability measures were identical to Experiment 1. Subjective workload was measured after each trial using the Air Traffic Workload Input Technique (ATWIT; Stein, 1985). Participants had 5 s to rate their workload from 1 (very low) to 10 (very high). ATWIT was used in Experiment 2 to obtain estimates of perceived workload after each trial.

Manual (unaided) training was identical to Experiment 1. Participants then watched a PowerPoint explaining low and high transparency and were notified what type of transparency they would receive during Block 1. They then completed Block 1. Participants then watched another PowerPoint specifying transparency during Block 2, followed by Block 2 trials. Finally, they watched a PowerPoint relevant to the transparency in Block 3, followed by Block 3 trials. After each block participants completed the trust and usability questionnaires (order counterbalanced) and took a break of at least 60 s. Total duration of the experiment was 2.5 h.

## Results

**Figure 7:**
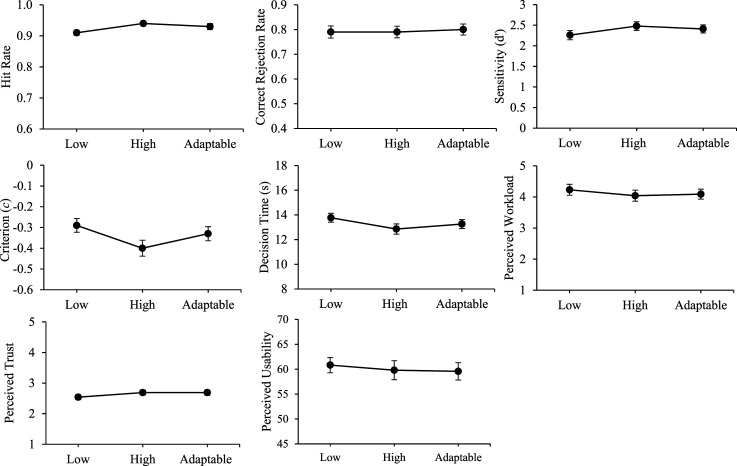
Accuracy of automation use, decision time, workload, trust, and usability as a function of transparency. *Note.* Error bars represent the standard error of the mean. Low = low transparency; high = high transparency; adaptable = adaptable transparency.

Descriptive statistics for test trials, split by transparency are presented in Table 5 and illustrated in Figure 7. We ran 3 Transparency (fixed low, fixed high, adaptable) × 6 Transparency Presentation Order (adaptable first and fixed low last, adaptable first and fixed high last, fixed high first and adaptable last, fixed high first and fixed low last, fixed low first and adaptable last, fixed low first and fixed high last) mixed ANOVAs to examine the effect of transparency on outcome variables. Main effects of transparency were followed up with planned paired samples *t*-tests that directly paralleled our research questions by comparing fixed low and high transparency, fixed low and adaptable transparency, and fixed high and adaptable transparency.

**Table 5.** Means and Standard Deviations (Presented in Parentheses) as a Function of Transparency.

	Fixed Low Transparency	Fixed High Transparency	Adaptable Transparency
Hit rate	.91 (.08)	.94 (.07)	.93 (.09)
CR	.79 (.23)	.79 (.22)	.80 (.21)
Sensitivity (*d′*)	2.26 (1.06)	2.48 (.99)	2.41 (.93)
Criterion (*c*)	−.29 (.31)	−.40 (.36)	−.33 (.32)
DT (s)	13.77 (3.37)	12.86 (3.89)	13.26 (3.40)
High transparency selection	-	-	.43 (.40)
Perceived workload	4.23 (1.65)	4.04 (1.66)	4.09 (1.49)
Trust	2.54 (.75)	2.69 (.76)	2.69 (.78)
Usability	60.82 (14.35)	59.80 (17.91)	59.57 (16.35)

*Note.* CR = correct rejection rate; DT = correct decision time; s = seconds.

Interactions between transparency and transparency presentation order were followed up with independent samples *t*-tests designed to directly assess the impact of the order of transparency presentation on the key aforementioned comparisons of interest. To do this, we compared fixed low and high transparency, fixed low and adaptable transparency, and fixed high and adaptable transparency, as a function of whether the conditions in each comparison had (both) been presented in Block 1 (first block) or Block 3 (the last block) (i.e. between-subject comparison).

### Adaptable Transparency Selection

During the adaptable transparency block, participants selected high transparency on 43% of missions, with considerable variation across participants (*SD* = .40). Twenty-one participants (23.9%) never selected high transparency, 12 (13.6%) selected it on every trial, while the remaining 55 (62.5%) used a mix of low and high transparency.

### Accuracy of Automation Use

#### Hit Rate

There was a main effect of transparency, *F* (2,164) = 9.90, *p* < .001, $\eta_{\rho }^{2}$ = .11, but not order, *F* < 1. Hit rates were higher with fixed high, *t* (87) = 4.29, *p* < .001, *d* = .46, and adaptable, *t* (87) = 2.30 *p* = .02, *d* = .25, compared to fixed low transparency. There was no difference in hit rate between the fixed high and adaptable transparency, *t* (87) = 1.96, *p* = .053.

There was an interaction between transparency and order, *F* (2,164) = 2.35, *p* = .01, $\eta_{\rho }^{2}$ = .13. Table 6 indicates hit rate improved in Block 3 compared to Block 1 across conditions, explaining the interaction between transparency and order. In Block 1, there were no differences in hit rate between any transparency condition, *t*s < 1. In Block 3, hit rates were higher for fixed high compared to fixed low transparency, *t* (56) = 2.40, *p* = .02, *d* = .63, with no difference between adaptable and fixed low, *t* (57) = 1.40, *p* = .17, or fixed high, *t* < 1.

**Table 6.** Means and Standard Deviations (Presented in Parentheses) for Outcome Variables With Significant Interactions Between Transparency and Transparency Presentation Order.

	Fixed Low First	Fixed High First	Adaptable First	Fixed Low Last	Fixed High Last	Adaptable Last
Hit rate	.91 (.09)	.92 (.05)	.91 (.09)	.91 (.10)	.96 (.05)	.94 (.08)
Sensitivity	2.24 (.88)	2.24 (.85)	2.08 (.96)	2.30 (.97)	2.85 (.85)	2.48 (1.00)
DT (s)	15.21 (3.40)	14.70 (3.14)	14.78 (4.02)	13.09 (2.90)	10.99 (3.44)	11.98 (3.08)
Usability	58.88 (12.98)	55.60 (15.59)	60.83 (16.91)	62.67 (14.28)	60.86 (21.04)	63.33 (15.54)

*Note.* Low = low transparency; High = high transparency; DT = correct decision time; s = seconds.

#### Correct Rejection Rate

There were no main effects of transparency, *F* < 1, or order, *F* < 1, and no interaction, *F* (2, 164) = 1.63, *p* = .10.

#### Sensitivity

There were no main effects of transparency, *F* (2,164) = 2.65, *p* = .07, or order, *F* < 1. There was an interaction between transparency and order, *F* (2,164) = 2.53, *p* = .01, $\eta_{\rho }^{2}$ = .13. Table 6 indicates that sensitivity improved in Block 3 compared to Block 1 across conditions, explaining the interaction between transparency and order. In Block 1, there were no differences in sensitivity between any transparency condition, *ts* < 1. However, in Block 3, sensitivity was higher for fixed high compared to fixed low transparency, *t* (56) = 2.30, *p* = .03, *d* = .60, with no differences between adaptable and fixed low, t < 1, or fixed high, *t* (57) = 1.55, *p* = .13.

#### Criterion

There were no main effects of transparency, *F* (2,164) = 3.13, *p* = .050, or order, *F* (1,82) = 1.12, *p* = .36, and no interaction, *F* (2,164) = 1.27, *p* = .25.

### Correct Decision Time

There was a main effect of transparency, *F* (2,164) = 4.71, *p* = .01, $\eta_{\rho }^{2}$ = .05, but not order, *F* < 1. Decisions were faster with fixed high compared to fixed low transparency, *t* (87) = 2.53, *p* = .01, *d* = .27. There were no differences in decision time between fixed low and adaptable, *t* (87) = 1.39, *p* = .17, or between the fixed high and adaptable transparency, *t* (87) = 1.02, *p* = .31.

There was an interaction between transparency and order, *F* (2,164) = 11.34, *p* < .001, $\eta_{\rho }^{2}$ = .41. Table 6 indicates faster decision times in Block 3 compared to Block 1 across conditions, explaining the interaction between transparency and order. In Block 1, there were no differences in decision time between any transparency condition, *t*s<1. However, in Block 3, decision times were faster for fixed high compared to fixed low transparency, *t* (56) = 2.51, *p* = .02, *d* = .66. There were no differences between adaptable and fixed low, *t* (57) = 1.43, *p* = .16, or fixed high transparency, *t* (57) = 1.16, *p* = .25.

### Perceived Workload, Trust, and Usability

There were no main effects of transparency on perceived workload, *F* (2,164) = 3.16, *p* = .050, or order, *F* (1,82) = 2.15, *p* = .07, and no interaction, *F* (2,164) = 1.87, *p* = .053.

For trust, there was a main effect of transparency, *F* (2,162) = 3.24, *p* = .04, $\eta_{\rho }^{2}$ = .04.

Trust was higher with adaptable compared to fixed low transparency, *t* (87) = 2.20, *p* = .03, *d* = .24. There was no difference in trust ratings between fixed high and fixed low, *t* (87) = 1.98, *p* = .051, *d* = .21, or between fixed high and adaptable transparency, *t* < 1. There was no main effect of order, *F* (1,81) = 1.36, *p* = .25, and no interaction, *F* (1,162) = 1.73, *p* = .08.

For usability, there were no main effects of transparency, *F* < 1, or order, *F* < 1. There was an interaction between transparency and order, *F* (2,164) = 2.80, *p* = .003, $\eta_{\rho }^{2}$ = .15. Table 6 indicates higher usability ratings in Block 3 compared to Block 1 across conditions, explaining the interaction between transparency and order. In Block 1, there were no differences in usability ratings between fixed low and fixed high, *t* < 1, fixed low and adaptable, *t* < 1, or fixed high and adaptable transparency, *t* (57) = 1.23, *p* = .22. In Block 3, there were no differences in usability between any transparency condition, *t*s<1.

## Aggregated Analysis of UV Management Task Fixed Transparency Effects

In Experiment 1 we did not observe a difference in the accuracy of automation use (sensitivity) or other outcomes with fixed high compared to low transparency. Following changes to the task design in Experiment 2 (i.e. removal of the surveillance phase and separation of fixed low and high transparency blocks) to bring it in line with our prior research, we still found no significant differences in sensitivity (note that the specific fixed high vs. low transparency comparison was not made because the omnibus test did not reach significance, *p* = .07), but there was evidence of faster correct decisions. There was a significant fixed high versus low sensitivity effect at Block 2 of Experiment 2. More generally, however, these findings do not replicate the outcomes of our prior studies that used the same or very similar UV management task (Tatasciore et al., 2023, 2024; Tatasciore & Loft, 2024, 2025).

**Figure 8.**
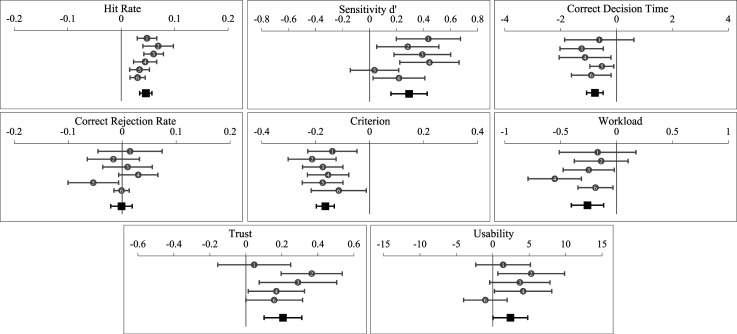
Aggregated analysis forest plots between fixed high and low transparency conditions on outcomes variables. *Note.* The grey circles represent the mean differences between the fixed high and low transparency conditions on outcome variables for (1) Tatasciore et al. (2023), (2) Tatasciore and Loft (2024), (3) Tatasciore et al. (2024), (4) Tatasciore and Loft (2025), (5) Current Study Experiment 1, and (6) Current Study Experiment 2. The black squares represent the aggregated analysis data point. 95% confidence intervals are presented. The Tatasciore & Loft (2024) (2) study is excluded from correct decision time as this study manipulated decision time. The current Study Experiment 1 (5) is excluded from workload, trust, and usability as fixed low and high transparency conditions were intermixed.

Given these inconsistencies between the current outcomes and our prior work, we conducted an aggregated analysis on the difference scores between the fixed high and low transparency conditions across outcome variables based on our UV management studies to date that manipulated fixed high versus low transparency (using Exploratory Software for Confidence Intervals; Cumming, 2012). A conservative random effect model was applied (as opposed to a fixed-effects model) to account for the heterogeneity across experiments, as well as variance in the individual effect sizes. Each experiment was weighted according to the inverse of the variance of its effect size (i.e. shorter confidence intervals signify larger aggregated analysis weight). Figure 8 presents forest plots showing mean effect sizes and 95% confidence intervals, and where appropriate the aggregated outcomes are discussed below.

Figure 8 indicates that on average across all our studies, fixed high transparency provision has led to significantly more accurate automation use (hit rate and sensitivity, but not correct rejection rate), faster correct decisions, lower perceived workload, and higher perceived trust and usability, compared to fixed low transparency. It has also led to an increased bias towards agreeing with automated advice. Notably for sensitivity, fixed high (compared to fixed low transparency) consistently has led to increased sensitivity across prior studies (Studies 1–4 in Figure 8), and the current Experiment 2 findings (Study 6 in Figure 8) were more consistent with that than the Experiment 1 findings (Study 5 in Figure 8), with the 95% confidence intervals for the high versus low fixed transparency effect size on sensitivity in Experiment 2 (Study 6 in Figure 8) not overlapping with 0. This reflects the fact that we changed the experimental design in Experiment 2 to closely match our prior work. The aggregated analysis indicated that the benefit of fixed high transparency on sensitivity has been driven by improved hit rates rather than improved correct rejection rates. This is also reflected by the increased automation response bias associated with fixed high transparency (Figure 8) that we discussed in the Introduction sections of this paper (Mosier & Skitka, 1996).

## General Discussion

The current study examined the extent to which adaptable automation transparency could improve the accuracy of automation use and other outcome variables compared to fixed low and high transparency. We examined factors underlying higher transparency selection (decision risk, perceived trial difficulty).

### Adaptable Transparency Selection

Despite there being no cost in terms of significant display clutter (Moacdieh & Sarter, 2015) or time to populate information displays, participants were relatively hesitant to select high transparency, engaging it on only 39% of trials in Experiment 1 and 43% in Experiment 2. The relatively low selection rates likely reflect that higher transparency requires more information to be processed, and that humans prefer to expend minimal cognitive effort to make decisions (Molloy & Parasuraman, 1996; Wickens & Hollands, 2000). In line with this interpretation, Experiment 1 indicated that participants selected high transparency for trials that they perceived as more difficult, indicating meta-cognition in monitoring the task environment to decide when higher transparency was required (Osman, 2010). Further, in Experiment 2 posttask questionnaires revealed that 63% of participants who selected high transparency at least once during the adaptable transparency block reported selecting high transparency based on perceived task difficulty (e.g. when they observed there were multiple relevant environmental factors, or when capability weighting combinations were harder).

Decision risk did not influence high transparency selection. This could be because participants perceived minimal decision risk. However, while we cannot rule this out, we think that it is unlikely because we informed participants that choosing the incorrect UV for high-risk missions would disproportionality decrease their monetary bonus. This was the same method of manipulating decision risk used by Loft et al. (2023), who reported increased automated advice verification and improved automated advice correct rejection rates with high decision risk in a similar UV management task.

### Effects of Adaptable Transparency on Outcomes

We had reasoned that adaptable transparency provision could lead to a net benefit to the accuracy of automation use because it would increase the probability that participants engage with and thoroughly process purposively selected high transparency information (Miller, 2023; Pescetelli et al., 2021), due to significant expected value of processing that information (Moray, 2003; Wickens et al., 2015). However, adaptable transparency did not improve the accuracy of automation use compared to fixed low or high transparency in Experiment 1 or 2. The only statistically reliable effect obtained was that perceived trust in automation was higher with adaptable compared to fixed low transparency in Experiment 2.

Overall, then, we found no reliable evidence that adaptable transparency improved the accuracy of automation use. While the aggregated analysis findings (Figure 8) indicate that although the lack of adaptable transparency advantage could have been caused by the lack of fixed transparency effects in Experiment 1, this is far less likely the explanation in Experiment 2 in which our fixed transparency effect (high vs. low) on sensitivity was far more closely aligned with our prior work. The potential negative impact of adaptable transparency on cognitive capacity does not appear a viable explanation because in Experiment 2 adaptable transparency did not increase decision time or perceived workload. We did find in Experiment 1 that perceived workload was higher with adaptable transparency. The difference across studies may be because in Experiment 1 workload was measured only once following the intermixed fixed low and high transparency block, as opposed to being measured separately for fixed low and high transparency blocks in Experiment 2 (and also measured more precisely in Experiment 2, after completion of each trial).

It is possible that participants in the current study did not have sufficient meta-cognitive skills/capacity to accurately monitor and estimate task trial difficulty or their own performance capabilities, resulting in lower than optimal high transparency-selection rates (Osman, 2010). Vered et al. (2020) reported that demand-driven transparency (i.e. adaptable transparency) conditions led to faster correct decision times, yet comparable decision accuracy to sequential transparency (i.e. where participants stepped through each level of transparency) conditions. In Vered et al. (2020), participants selected high transparency on 68% of trials, compared to only 41% of trials on average across the current two experiments. Although the current findings indicate high transparency was selected for more difficult trials, we could not meaningfully determine whether the accuracy of automation use improved as a function of high transparency selection in adaptable blocks given that selection rates varied considerably across participants, and the accuracy of automation use also likely varied with objective trial difficulty (i.e. high transparency was likely selected on more difficult trials). Thus, we hesitate to conclude that high transparency was under-selected. Future research could examine whether providing a trial (mission) difficulty metric, potentially adapted in real-time based on each participant’s historical manual performance across missions, could facilitate the meta-cognition required to purposefully select high transparency information, and whether that design feature can lead to a net benefit to the accuracy of automation use with adaptable transparency.

### Limitations and Conclusions

Although the UV task used here broadly represents contexts where operators receive decision support, it does not include the same level of information complexity and multi-tasking requirements of real UV management. In addition, we used novice participants whose skill acquisition on the UV task was trained but did not reach plateau, indicated by the practice effects observed (i.e. transparency by transparency presentation order interactions). Thus, it remains possible that experts may employ different adaptable transparency use strategies. Despite significant challenges in achieving adequate experimental control and sufficiently sized expert participant samples, future research could examine the effects of adaptable transparency with experts using higher-fidelity simulations. Further, despite no reliable evidence for apparent meaningful transparency presentation order effects on our statistical comparisons of interest, we cannot rule out the fact that our failure to replicate the fixed transparency effects from our prior studies was due to the inclusion of the adaptable transparency in the current within-subjects designs. Our automated advice reliability was relatively high (80%), and future studies should examine the potential moderating effect of reliability on high transparency selection and adaptable transparency outcomes. High transparency is likely to be selected more under conditions of lower automation reliability.

In conclusion, adaptable transparency did not improve the accuracy of automation use compared to fixed low or high transparency in either experiment. In addition, fixed high transparency failed to significantly improve the accuracy of automation use (sensitivity) or other outcome variables in either experiment compared to fixed low transparency. The aggregated analysis however clearly indicated that the fixed transparency effect on sensitivity in Experiment 2 was closely aligned with our prior work. Regardless, adaptable transparency did not improve the accuracy of automation use compared to fixed low or high transparency in Experiment 2. It is crucial that such null findings are published to avoid the potential ‘file drawer’ problem (Pashler & Wagenmakers, 2012), and this is particularly vital when knowledge could be used by practitioners (Jones et al., 2010). Aggregated analysis of transparency design interventions should include all results obtained, not just the positive cases (Cumming, 2012). Synthesizing findings from multiple studies can be used to derive more comprehensive conclusions based on more precise effect size estimates and in doing so facilitates evidence-based system design decision making.

## Key Points

• Examined whether allowing participants to select high transparency (adaptable transparency) could improve their accuracy of automation use compared to fixed low and high transparency.
• Participants completed an uninhabited vehicle (UV) management task aided by imperfect automation that advised the optimal UV to complete missions.
• With adaptable transparency, participants selected higher transparency on 41% of missions and were more likely to select high transparency for missions they perceived as more difficult.
• There was no evidence that adaptable transparency improved automation use compared to fixed transparency.
• Despite a lack of fixed transparency effects, an aggregated analysis of findings to date using this UV management task indicated that higher fixed transparency improves automaton use and other outcome variables.
